# Evaluation of a translational swine model of respiratory hypersensitivity induced by exposure to *Phleum pratense* pollen allergens

**DOI:** 10.3389/falgy.2025.1557656

**Published:** 2025-04-07

**Authors:** Jessica Ledesma-Aparicio, Gustavo Salazar-Guerrero, Axel Soto-Muñoz, Carmen Ramírez-Estudillo, Mónica Luz Gómez-Esquivel, Juan Pablo Reyes-Grajeda, César A. Reyes-López, Marco A. Vega-López

**Affiliations:** ^1^ Laboratorio de Estructura de Proteínas, Instituto Nacional de Medicina Genómica (INMEGEN), Mexico City, Mexico; ^2^ Laboratorio de Bioquímica Estructural, Escuela Nacional de Medicina y Homeopatía del Instituto Politécnico Nacional, Mexico City, Mexico; ^3^ Departamento de Infectómica y Patogénesis Molecular, Cinvestav, Zacatenco, Mexico City, México

**Keywords:** asthma, allergy, minipig, pollen, hypersensitivity

## Abstract

**Introduction:**

Asthma is a disease characterized by chronic inflammation of the airway mucosa that causes tissue remodeling and a reversible decrease in airflow. The causative agent of asthma is still unknown; however, several studies have shown that environmental factors such as allergens present in pollens are involved. This project's objective was to develop and evaluate a model of respiratory hypersensitivity in Vietnamese minipigs, which is closer in many aspects to humans than rodents, using *Phleum pratense* allergenic pollen extract.

**Methods:**

In this hypersensitivity model, human-like signs were observed during a challenge with the allergens. Intradermal and passive anaphylaxis tests confirmed that specific IgE mediated the response.

**Results:**

Significant changes in lung tissue remodeling, high levels of serum allergen-specific IgA, IgG, and to a lesser extent IgE were found in the sensitized pigs, which could favor tolerance and pathogenesis. However, since chronic pathology did not develop, elevated levels of cytokines were not proven.

**Discussion:**

This work demonstrated that the immunization protocol in this experimental model can induce a type I respiratory hypersensitivity-like response mediated by antigen-specific IgE, with pathophysiological similarities to those of humans and prospective for translational basic and applied research.

## 1 Introduction

Asthma is a disease characterized by chronic airway mucosal inflammation and bronchial hyperresponsiveness, which results in tissue remodeling and reversible airflow reduction (1). Asthma has diverse clinical manifestations, highlighting episodes of repeated shortness of breath and wheezing, which are at least partially reversible, recurrent cough, and excess airway mucus production (2). The World Health Organization (WHO) estimates that this disease affects 262 million people and currently causes 455,000 asthma deaths per year (3), with a prevalence of 15% in first-world countries, while studies in Latin America have revealed figures ranging from 5.7%–16.5% in the population (4).

Clinical and laboratory data show asthma can be classified as nonallergic (intrinsic) or allergic (extrinsic). Non-allergic asthma is typically present in adulthood and is more severe but is not associated with elevated serum allergen-specific IgE titers, as it is often associated with nasal polyposis and rhinitis (5). Allergic asthma usually begins in childhood and is characterized by dependence on a constant allergen stimulus, positive skin tests, and elevated serum allergen-specific IgE titers. At present, the precise pathogenesis of allergic asthma is still unknown, but several studies have shown that environmental factors such as allergens are involved (6). Allergens are perceived by the body as harmful, which triggers an exaggerated reaction of the immune system, aeroallergens such as grass pollen are one of the main triggers of asthma exacerbations (7). Grass pollen from *Phleum pratense* is one of the most widespread grasses in the world and has been linked to allergic processes, asthma, and even hay fever (8). It is estimated that 80% of asthmatic children and 40%–50% of asthmatic adults have allergic mechanisms caused by allergens (9).

Currently, experimental animal models of asthma, primarily developed in mice and rats, have become an indispensable tool for histological and molecular research on this disease; however, due to structural differences as well as the size of the organs compared to humans, it has not been possible to deepen research where, for example, the drugs and allergen-specific immunotherapy dosages are not easily extrapolated from these animal models to humans (10–13). Pigs are most like humans in terms of anatomy, immunology, biochemistry, physiology, size, and genetics (14–18). The pig has been used as an animal model for cystic fibrosis, as porcine lungs have marked similarities to humans in tracheobronchial tree structure, lung physiology, airway morphology, abundance of airway submucosal glands, and glycoprotein synthesis patterns, allowing the model to be translational for various pathologies present in humans (18). Therefore, this model would help to elucidate some main unknowns of asthma, for example, to identify the early pathogenesis of the disease, the kinetics of the humoral response from the first contact with the antigen to the development of sensitization where the antibody response changes from classic IgG and IgA to an IgE profile, to show how tissue remodeling is generated, including the molecular mechanisms by which the allergen crosses the mucosal barrier, etc.

The porcine hypersensitivity model would allow testing new asthma treatments and desensitization protocols, making the dosage extrapolation easier due to its similar weight and size to humans (19, 20). Surprisingly, the pig has been used as a model for asthma only in limited instances, mainly using ovalbumin sensitization models, leaving aside the use of clinically relevant allergens in the development of asthma (21).

Hence, this research aimed to validate the porcine model of respiratory hypersensitivity to a clinically relevant human allergen such *Phleum pratense*, using a novel sensitization protocol to induce the early development of the serum and mucosal response to the allergen, evaluating key clinical signs and features of an IgE mediated response to elucidate the suitability of the model for the study of asthma.

## 2 Materials and methods

### 2.1 Animals

Thirty-two weaned male and female specific pathogen-free (SPF), Vietnamese mini-pigs (28 days of age) from the Experimental Animal Production Unit (UPEAL-Cinvestav) were used. They were provided *ad libitum* access to food and water and maintained in conditions under Mexican government regulations (NOM-062-ZOO-1999). The Institutional Committee for the Care and Use of Laboratory Animals (CICUAL) of Cinvestav approved the experimental protocol (0143-15).

### 2.2 Allergen sensitization protocol

The allergenic antigens came from a standardized protein extract of *Phleum pratense* (Standardized Timoty Grass, Alk Abello Pharm, USA, 100,000 BAU/ml, Lot 0001638321). Aluminum hydroxide [Al (OH)_3_] 64 mg (4.0 g/100 ml), (Alergel, suspension, 431M98 SSA, ARLEX Mexico, S.A. de C.V.) was used as an adjuvant. The immunization protocol was a modification of a pig allergen-induced lung inflammation (21) and a mouse respiratory system sensitization protocol (22). Pollen-treated animals received 1 mg of total protein from allergenic pollen extract+64 mg aluminum hydroxide per dose and control animals received the adjuvant and phosphate solution (PBS) only. The animals were divided into the following experimental groups (*n* = 32): Experiment I: Five pigs were immunized to evaluate infiltrating cells in the intradermal and mucosal areas and 6 control pigs; experiment II: Eleven immunized pigs to assess clinical signs and the cytokine profile at different times and 10 control pigs. Two additional pigs were used for IgE production and purification, to immunize mice to obtain porcine anti-IgE antiserum.

**Figure 1:**
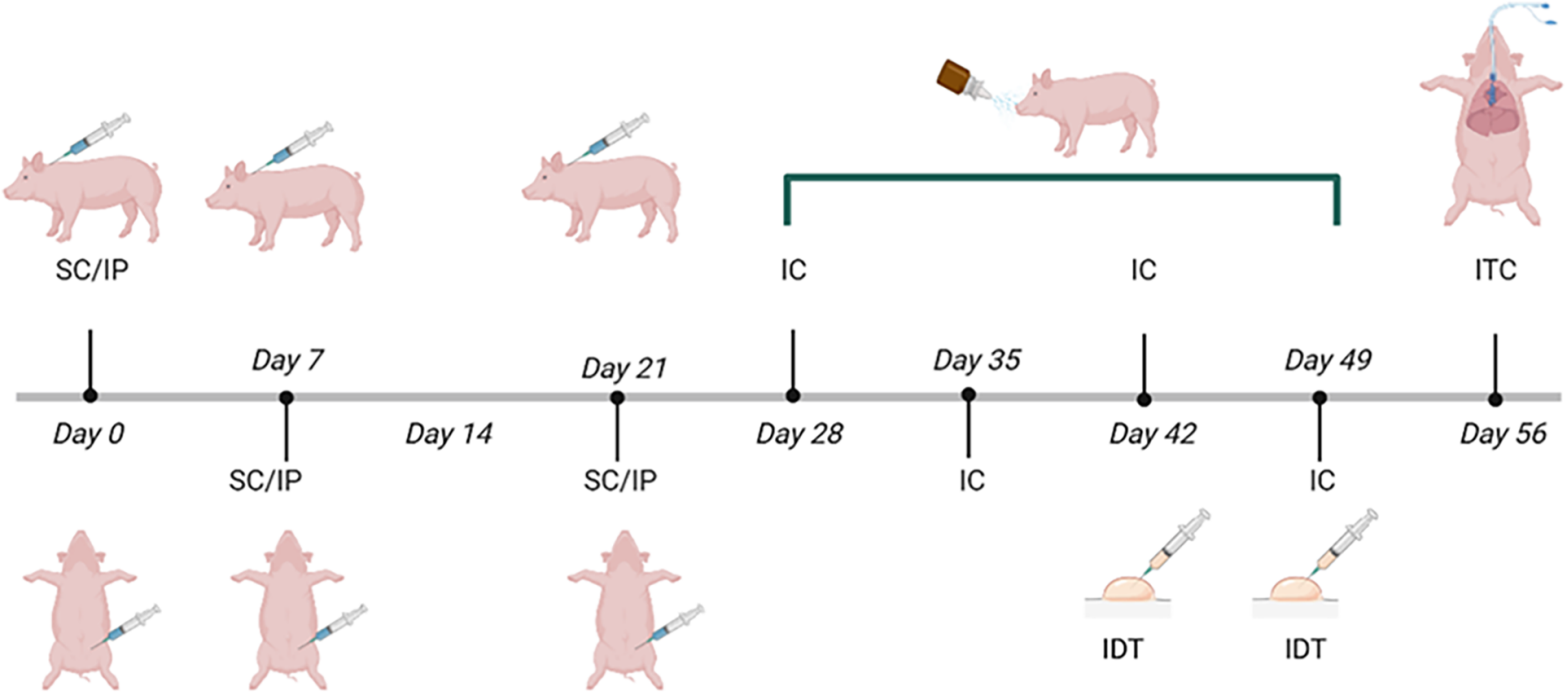
Immunization scheme with *Phleum pratense* pollen extract in Vietnamese minipigs. The onset of immunization (day 0) was at 28 days of age. The dose [1 mg proteins from pollen +64 mg Al(OH)_3_] was administered subcutaneously (SC) behind the ear and intraperitoneally (IP) on days 0, 7, and 21, intranasal challenges (IC) were performed on days 28, 35, 42 and 49. Intradermal Test (IDT) was performed on days 42 and 49. An intratracheal challenge (ITC) was carried out on day 56. Control groups only received adjuvant [64 mg Al (OH)_3_ + PBS].

The routes of administration varied as the experiment progressed, as shown in the immunization scheme (Figure 1). The immunization protocol started on day 28 of birth (one week after weaning). Pigs were humanely euthanized at the end of the protocol.

### 2.3 Subcutaneous, intraperitoneal, and intranasal immunizations

The dose [1 mg of proteins from pollen +64 mg Al (OH)_3_] was administered both subcutaneously (SC) behind the ear and intraperitoneally (IP), with subsequent intranasal (IN) challenges, based on a modification of the protocol reported by Mitchell et al. (21) and our previous experience with mucosal immunization (23–25). After immunization, vital signs, including heart and respiratory rates, were monitored to detect possible adverse effects such as discomfort or anaphylaxis. In addition, the application site was monitored for induration, redness, or itching. Control pigs received only the adjuvant [64 mg Al (OH)_3_ + PBS] under the same conditions. A total of 3 immunizations were applied on days 0, 7, and 21 (Figure 1). Intranasal instillations were performed with a 1 ml needleless syringe. Pollen-immunized pigs received a dose of 100 *μ*g of pollen extract adjusted to a final volume of 500 *μ*l with PBS, control pigs were instilled with 500 *μ*l of PBS.

### 2.4 Intradermal testing and biopsy

The ventral area of the pigs was divided into quadrants to locate the area of application. PBS (200 *μ*l) was administered as a negative control in one quadrant and pollen extract (100 *μ*g of extract in 200 *μ*l of PBS) in another. The test was read about 15 min post-application. A classical positive response was identified by erythema and induration. Skin biopsy collection with a sterile punch (Harris, Uni-Core 8.00 No. 7093508) was taken at the intradermal test site in all experimental animals after anesthesia with intramuscular (IM) Azaperone (2 mg/kg) (Sural, Laboratorios Chinoin. Mexico) and intravenous (IV) Tiletamine-Zolazepam (4.2 mg/kg) (Zoletil, Laboratorios Virbac, Mexico). Control pigs were tested under the same conditions 24 h before euthanasia to avoid prior pollen extract exposure. The intradermal test and skin biopsy were adapted and modified in our experimental model based on that previously described by Raszyk (26) and Nischal et al. (27).

### 2.5 Intratracheal challenge

The intratracheal challenge was performed in groups I and II using an orotracheal tube (Portex, oral/tracheal siliconized, 4.0 mm, Smiths Medical International Limited, UK), prior anesthesia and sedation with Azaperone (2 mg/Kg) and Tiletamine-Zolazepam (4.2 mg/Kg). The probe was introduced until reaching the bifurcation of the trachea and pollen extract (2 mg in 3 ml of PBS) was administered. At the end of the procedure, the probe was carefully removed, and the pigs were kept under constant surveillance and monitoring of signs (heart and respiratory rate) until their complete recovery. The procedure was adapted to our porcine respiratory hypersensitivity model based on that previously described by Nienhoff (28) and Judge et al. (18).

### 2.6 Passive cutaneous anaphylaxis (PCA) test

Sera from the pigs immunized with pollen proteins were collected, and an aliquot of each serum was inactivated at 56°C for 4 h, to determine whether the immune response was IgE mediated. Under the same conditions, sera from pigs unrelated to the project and PBS were used as negative controls. For the PCA test, two weaned pigs not included in the immunization protocol were employed. A volume of 200 *μ*l of each test, and control sera were intradermally injected at different well-identified sites distributed along the ventral area of each animal. After 20 h, 1 mg/kg body weight of pollen extract was administered intravenously. A positive reaction, characterized by erythema and induration, was observed in about 15 min at the injection site (29, 30).

### 2.7 Sample collection

Blood, saliva, and nasal mucus samples were collected. A comprehensive review of the available literature was conducted to ascertain the optimal sampling times for Group II (31–33). Samples were taken before (time 0) and after immunization (5 and 24 h), to ensure the detection of cytokines released after the stimulus.

Blood samples (3–5 ml) were obtained by puncture in the jugular vein, these samples were centrifuged at 4,000 RPM to obtain serum, which was then aliquoted into microtubes and stored at −80°C until further analysis. Nasal mucus and saliva samples were collected using cotton swabs and kept on ice, for subsequent centrifugation at 4,000 RPM for 10 min. Protease inhibitors (PMSF, TPCK, and TLCK) were added to the mucosal sample for proper preservation, aliquoted, and stored at −80°C, as described elsewhere (23).

### 2.8 Euthanasia

All the animals in the experiment were humanely euthanized by prior anesthesia with intramuscular Azaperone (2 mg/kg) and intravenous Tiletamine-Zolazepam (4.2 mg/kg) until fully unconscious, followed by exsanguination severing the major blood vessels in the neck (carotid arteries and jugular veins) to induce rapid bleeding, accordingly with our protocol 0143-15, approved by our CICUAL.

### 2.9 Bronchoalveolar lavage (BAL) and determination of cellular infiltrates in the respiratory tract

After euthanasia, the lungs and trachea were dissected, pre-washed to remove residual blood, and processed as previously described (34). Briefly, the bronchoalveolar lavage (BAL) was obtained by ligating the trachea inserting a catheter in the first bronchial bifurcation and instilling 50 ml of PBS. The collected BAL fluid was centrifuged at 2,000 RPM for 10 min at 4°C. Cell counting was performed with an automated counter (Countess Automated Cell Counter C10227, Invitrogen, Carlsbad, CA. USA). Finally, the samples were diluted to obtain a total of 150,000 cells in a final volume of 80–100 *μ*l and cytocentrifugation was performed at 2,000 rpm/10 min at room temperature (Cell Preparation System Cytospin 3, Shandon No. 7400001, USA), stained with Kwik Diff reagent (Thermo Fisher Scientific Kit Kwik Diff No. 9990700, USA), and a differential cell counting was performed with optical microscopy.

### 2.10 Morphological and histopathological analysis

Macroscopic morphological analysis of the lungs was performed to identify tissue damage, analyzing the area by planimetry using photographs of the dorsal and ventral views of the lungs with a computer-assisted image analyzer (Image J ver 1.54 h). Cuts in the lung were made at the level of the bronchus in the left lung parenchyma and another longitudinal cut from the apical to the basal zone. Lung samples and skin biopsies were fixed in 4% formalin for 24 h or in Carnoýs fixative for 16 h. The tissues were paraffin-embedded, cut in a microtome at 3 *µ*m thick, and stained with toluidine blue (mast cells) or chromotrope 2R (eosinophils). The samples were observed under light microscopy (Nikon Eclipse E400*,* Japan) and the density of cells was recorded using a computer-assisted image analyzer (Image Pro Plus 7.0 software). The technique was based on our own experience (23) and the protocol described by Gandjeva et al. (35).

### 2.11 Determination of cytokines

Serum cytokine levels for TGFβ, IL-4, IL-6, IL-10, and IFNγ were determined by ELISA assays at 0, 5, and 24 h post intratracheal challenge. A commercial multiplex ELISA Kit (Porcine Cytokine Magnetic Bead Panel, Milliplex®) was used, at 1/5 serum dilution for TGFβ and neat samples for IL-4, 6, 10, and IFNγ. A commercial ELISA kit (Porcine IL-13, Ray Biotech, Inc.) and a 1/4 sample dilution was used for IL-13 determination. In all cases, the procedure recommended by the manufacturer of each kit was followed.

### 2.12 Obtention and characterization of porcine IgE

Two independent adult mini pigs were subcutaneous and intraperitoneally immunized at days 0, 7, 14, and 28 with *Taenia hydatigena* parasitic protein extract (746 *μ*g/ml of *Taenia hydatigena* protein extract +Al (OH)_3_, kindly provided by Dr. Fernando Alba-Hurtado (FES-Cuautitlan-UNAM, Edo. de Mexico) to induce reaginic antibody production. The serum was obtained at the end of the experiment (day 35) after the animals were humanely euthanized. The serum was threefold precipitated with a 33% saturated ammonium sulfate solution (36, 37). The purity was verified by 12% polyacrylamide gel electrophoresis. Porcine anti-IgM (Clone 5C9) and anti-IgA (Clone F9) mice monoclonal antibodies were coupled to cyanogen bromide-activated Sepharose (CNBr-Activated Sepharose 4 Fast Flow GE Healthcare, Germany), and a Protein G column (Protein G Sepharose®, Fast Flow, Germany) coupled with Sepharose, were used to remove porcine IgM, IgA and IgG, respectively. IgE characterization was performed using native SDS-PAGE and Western blot techniques to confirm purity and molecular weight identification (38).

Subsequently, purified IgE bands were excised from polyacrylamide gels for immunization of BALB/c mice according to standardized protocols to obtain porcine anti-IgE mouse antiserum (39, 40). The optimal dilution of the mouse porcine anti-IgE antiserum was determined by serial dilution using a pre-immune serum as a reference and comparing optical densities. The specificity of the antiserum was evaluated by Western blotting and IgE was specifically identified. Two weaned pigs that were not part of the protocol were used for the PCA test as previously described in this study.

### 2.13 Quantitative determination of allergen-specific IgA, IgG, and IgE

Antigen-specific ELISA assays were performed to measure IgA, IgG, and IgE concentrations in pig serum, saliva, and nasal mucosal samples. Briefly, 96-well ELISA microplates were sensitized with 0.2 *μ*g (2 *µ*g/ml) of the commercial extract of *Phleum pratense* per well. For immunodetection, peroxidase-coupled anti-porcine IgG and IgA antibodies (goat anti-Pig IgG-Fc Fragment Antibody Affinity Purified, A100-104 and goat anti-Pig IgA Antibody Affinity Purified, A100-102, Bethyl, USA), were used by the protocols previously described for this technique and our patented method (22, 23, 25, 41, 42).

Previously, a linear regression analysis was performed to compare the commercial porcine IgE standard with the one obtained in this study. The optimal conditions for antigen coating concentration (*Phleum pratense*) per well were also determined. IgE quantification was performed with the mouse anti-pig IgE antiserum obtained in this work at a 1/500 dilution, for immunodetection an anti-mouse IgG antibody coupled to HRP was used at a 1/1,500 dilution (HRP Goat anti-mouse IgG minimal x-reactivity BioLegend Cat 405306, USA). The 3,3′,5,5,5,5′-tetramethylbenzidine (TMB) (Sigma-Aldrich®) was used as substrate and the absorbance was read at 450 nm after adding 1M HCl to stop the peroxidase reaction.

## 3 Results

### 3.1 Intradermal reaction (IDR)

**Figure 2:**
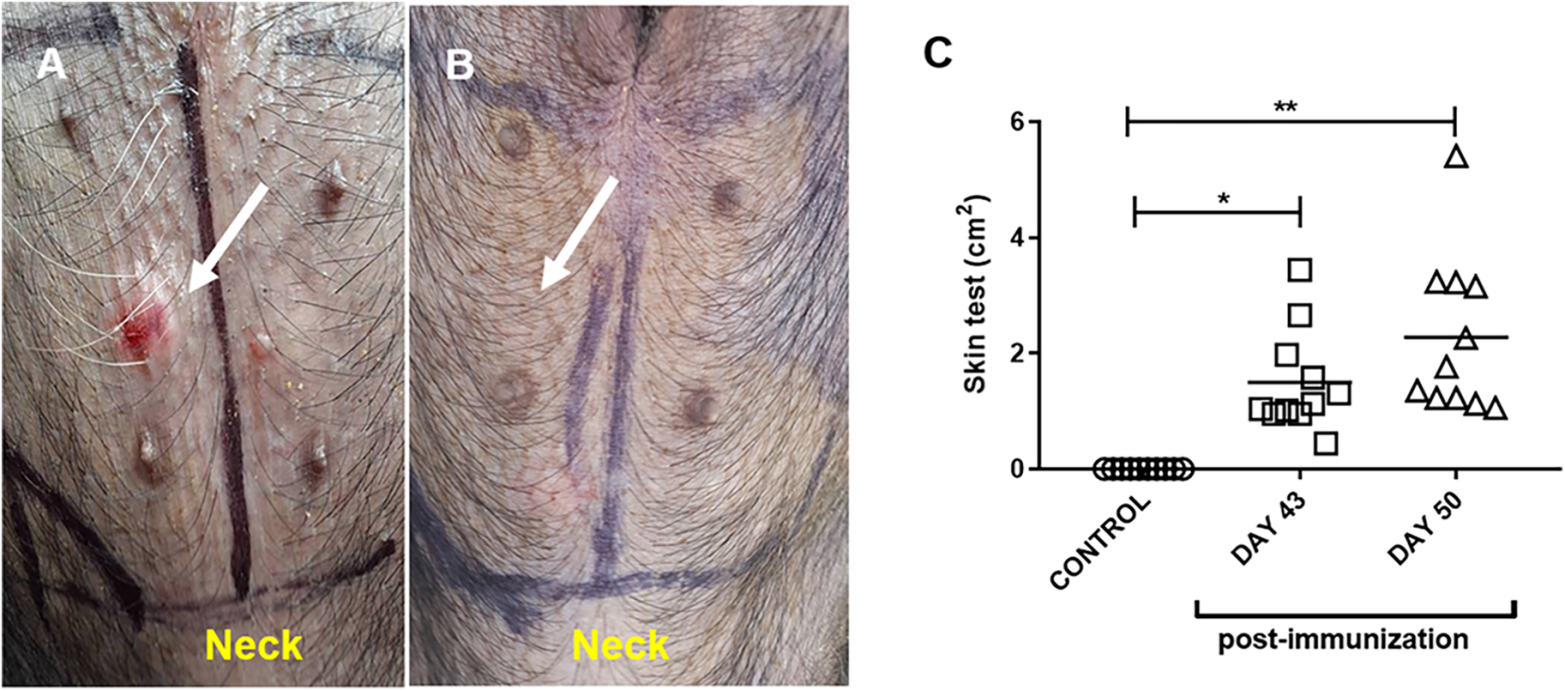
Representative intradermal test in pollen-sensitized **(A)** and control (adjuvant-only treated) **(B)** pigs. The arrow indicates the area of ID induration after 15 min of inoculation. **(C)** Area of erythema and induration in control (adjuvant) and pollen-sensitized (day 43 and day 50 PI) pigs after pollen inoculation. Each symbol represents one pig in two independent experiments. Horizontal bars represent the mean (*n* = 11). Significance calculated by Unpaired *t*-test. *p ≤ 0.05, **p < 0.01.

All the pollen extract-sensitized pigs were positive for the intradermal test performed on days 43 and 50 of the protocol (Figure 2A), the control group did not show any reaction (Figure 2B). The positive test was characterized by a rapid erythema reaction (observed within approximately 15 min) and, in some cases, induration in the areas tested with the pollen extract (Figure 2C).

### 3.2 Passive cutaneous anaphylaxis (PCA) test

**Figure 3:**
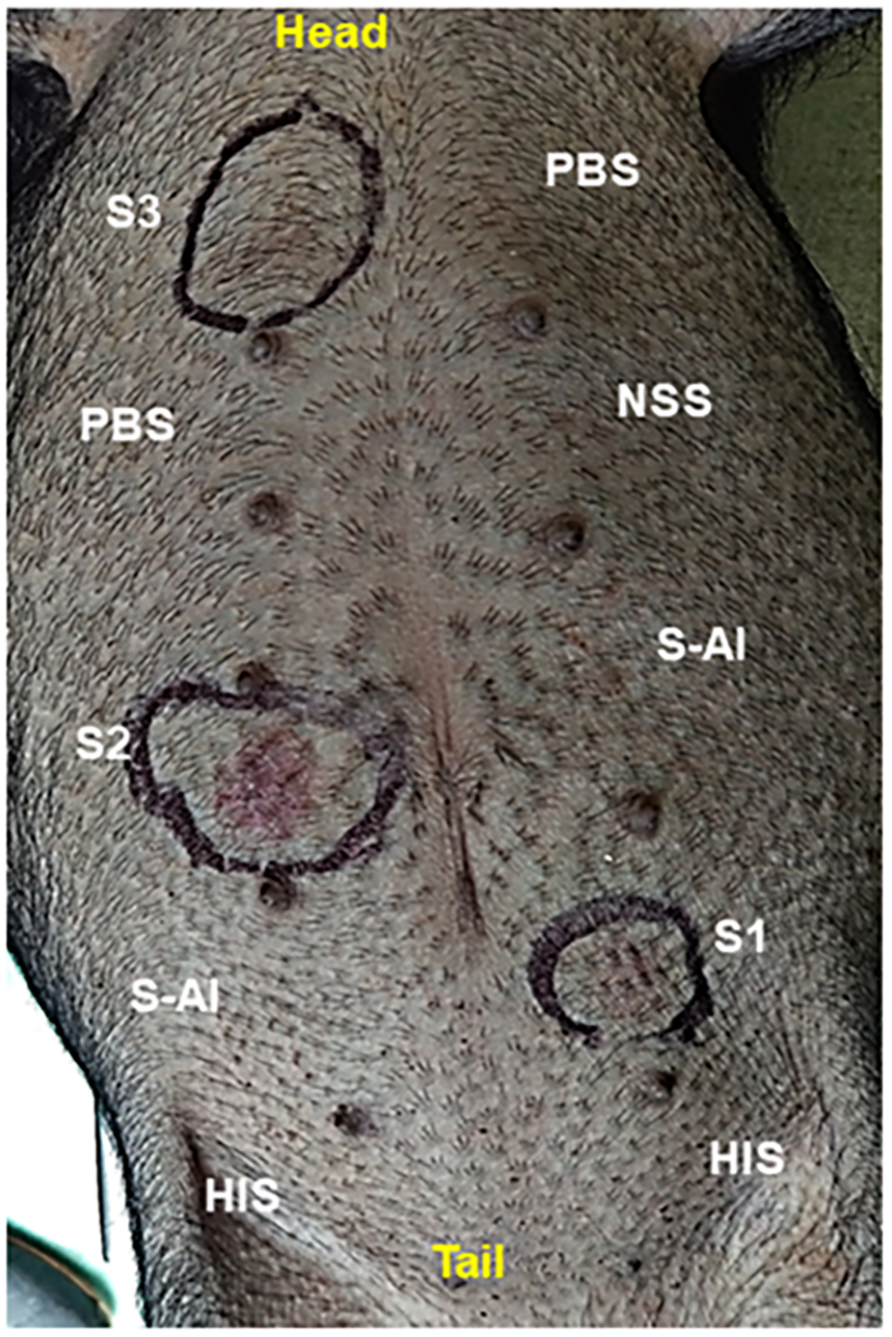
Passive cutaneous anaphylaxis (PCA) reaction in a naive non-sensitized pig. S1, S2, and S3 = fresh sera from pollen-sensitized pigs; HIS = heath-inactivated pollen-positive serum; NSS = non-sensitized pig serum; S-Al = pig control serum; PBS = inoculation control. After 24 h of the ID inoculation, the pig received intravenously 1 mg/Kg of pollen extract. An erythematous reaction (encircled) was evident within 15 min after administration.

Sensitization to pollen extract was confirmed by a passive cutaneous anaphylaxis (PCA) test, to evaluate whether the hypersensitivity response was mediated by specific IgE against pollen. Only positive responses were seen with fresh sera from sensitized pigs, presenting erythema within a few minutes after the test. Heat-inactivated positive sera gave a negative result, showing that the reaction was due to thermo-sensitive specific IgE for pollen. Fresh and inactivated sera from control pigs gave a negative PCA reaction (Figure 3).

### 3.3 Intratracheal challenge

**Figure 4:**
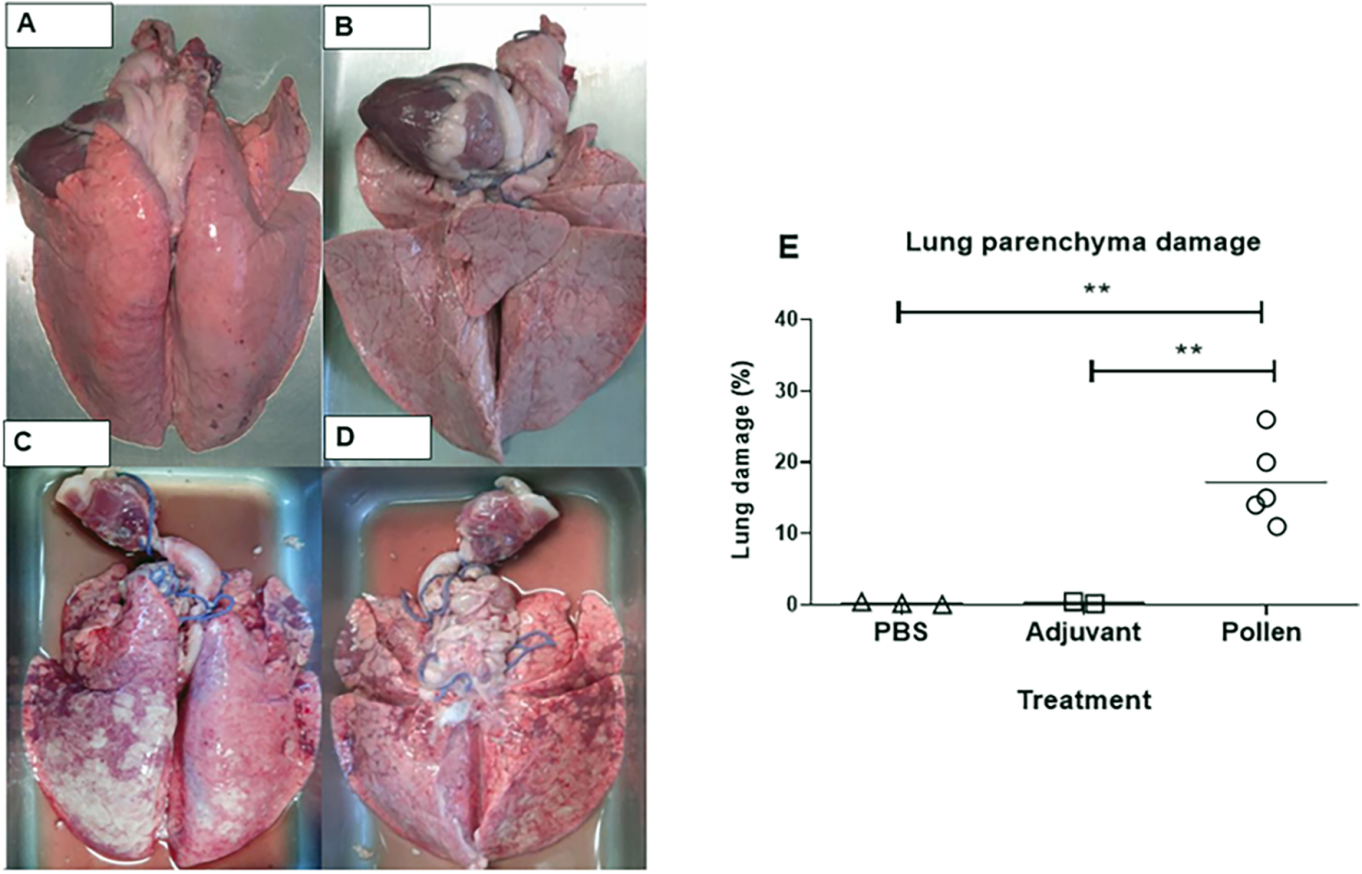
Representative dorsal and ventral images of lungs from control **(A,B)** and pollen-treated **(C,D)** pigs. **(E)** Percentage of tissue damage in lungs of control (PBS and Adjuvant, *n* = 3 each) and experimental (*n* = 5) pigs. Each figure represents one pig. Significance calculated by Unpaired *t*-test. **p ≤ 0.01.

In pigs immunized with pollen, clinical signs of anaphylactic reaction (bronchospasm, bilateral wheezing, intercostal twitching, peripheral cyanosis) were observed within minutes after the orotracheal administration of pollen extract. In one of the sensitized pigs, anaphylactic shock led to death (Table 1). The macroscopic morphology of the lungs of the pollen-treated pigs was analyzed and the presence of reddish multifocal stippling in different areas of the organ (petechial hemorrhages) was observed, in addition to the presence of tissue consolidations. The damage represented an average of 18% of the total visible area in both (ventral and dorsal) sides of the lung, finding a higher incidence of deterioration in the cranial and intermediate lobes with a significant difference (*p* ≤ 0.01) compared to the other experimental conditions (Figure 4). No tissue damage was observed in the control groups.

**Table 1:** Clinical signs before and after intratracheal challenge in experimental groups.

Group	Pig ID	Basal signs		Signs after intratracheal challenge		Observations
		Respiratory frequency/min	Heart rate/min	Respiratory frequency/min	Heart rate/min	
Pollen	30	44	84	65	75	• Anaphylactic shock • Bronchospasm • Bilateral wheezing • Intercostal retractions • Peripheral cyanosis • Dead Rescue therapy:dexamethasone, diphenhydramine
	31	48	116	55	70	
	32	45	112	60	72	
	36	39	104	80	70	
	37	43	116	40	60	
	38	44	85	40	50	
Control	33	33	96	43	90	Transient crackles
	34	34	87	55	80	
	35	35	90	80	75	
	39	39	95	40	72	
	40	40	105	60.	68	

### 3.4 Cellular infiltrates in bronchoalveolar lavage (BAL)

**Figure 5:**
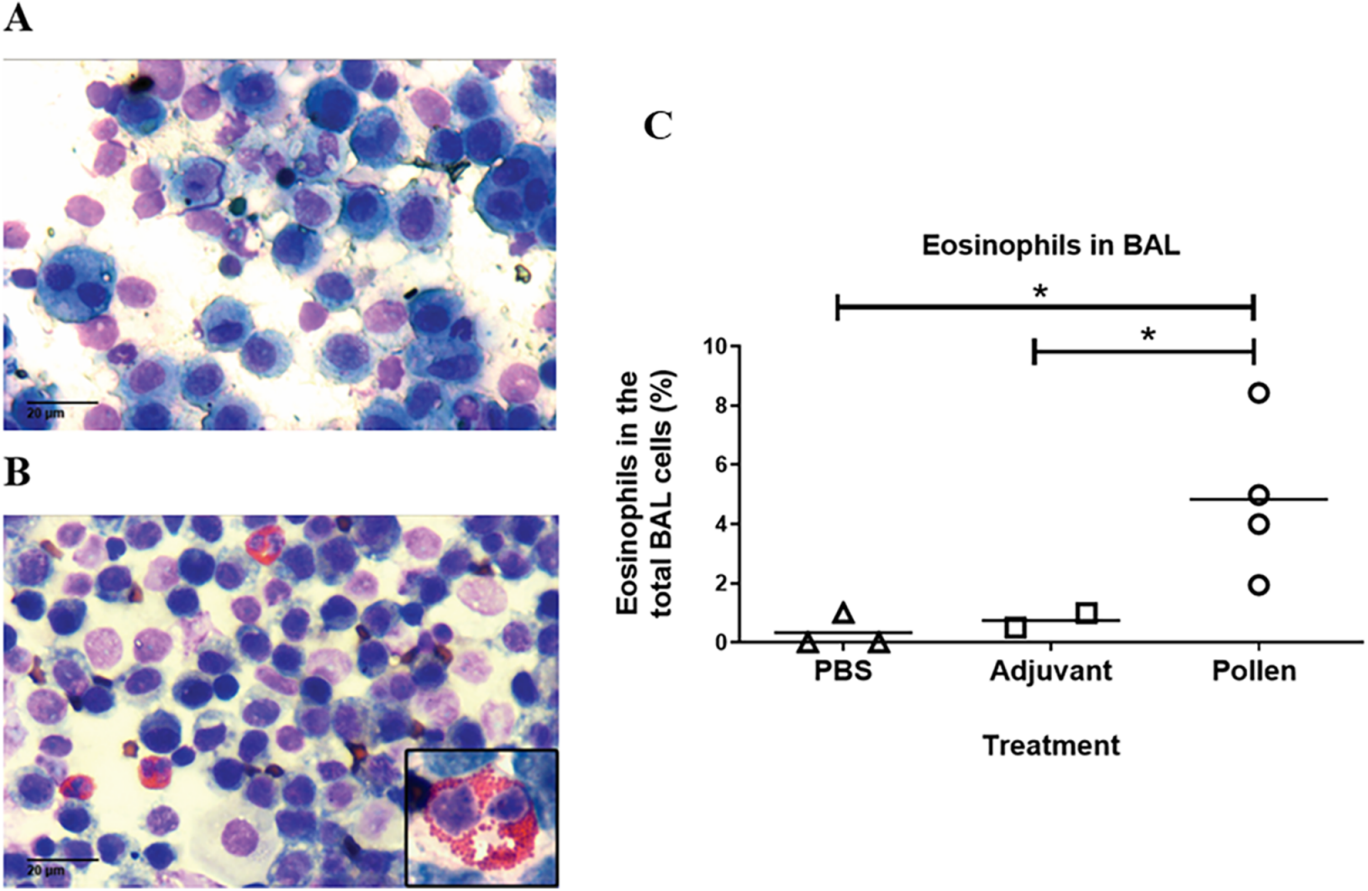
Representative bronchoalveolar lavage (BAL) in control **(A)** (PBS) and pollen-sensitized **(B)** pigs (40X, inset 100X). **(C)** Eosinophils in BAL from control (PBS and adjuvant) and pollen-sensitized pigs Each symbol represents an animal. Significance calculated by Unpaired *t*-test. *p ≤ 0.05.

The bronchoalveolar lavage (BAL) allowed us to identify the different cell populations in each experimental condition on a proportional and representative basis. In control pigs, macrophages and epithelial cells were normally found whereas in the pigs immunized with pollen, in addition to the presence of these cells, a significant increase in eosinophils was demonstrated (Figure 5), suggesting an inflammatory reaction caused by bronchial hyperreactivity and tissue remodeling. In contrast, the percentage of lymphocytes, neutrophils, and mast cells was not significantly different from control pigs (data not shown).

### 3.5 Histopathological analyses of lungs

**Figure 6:**
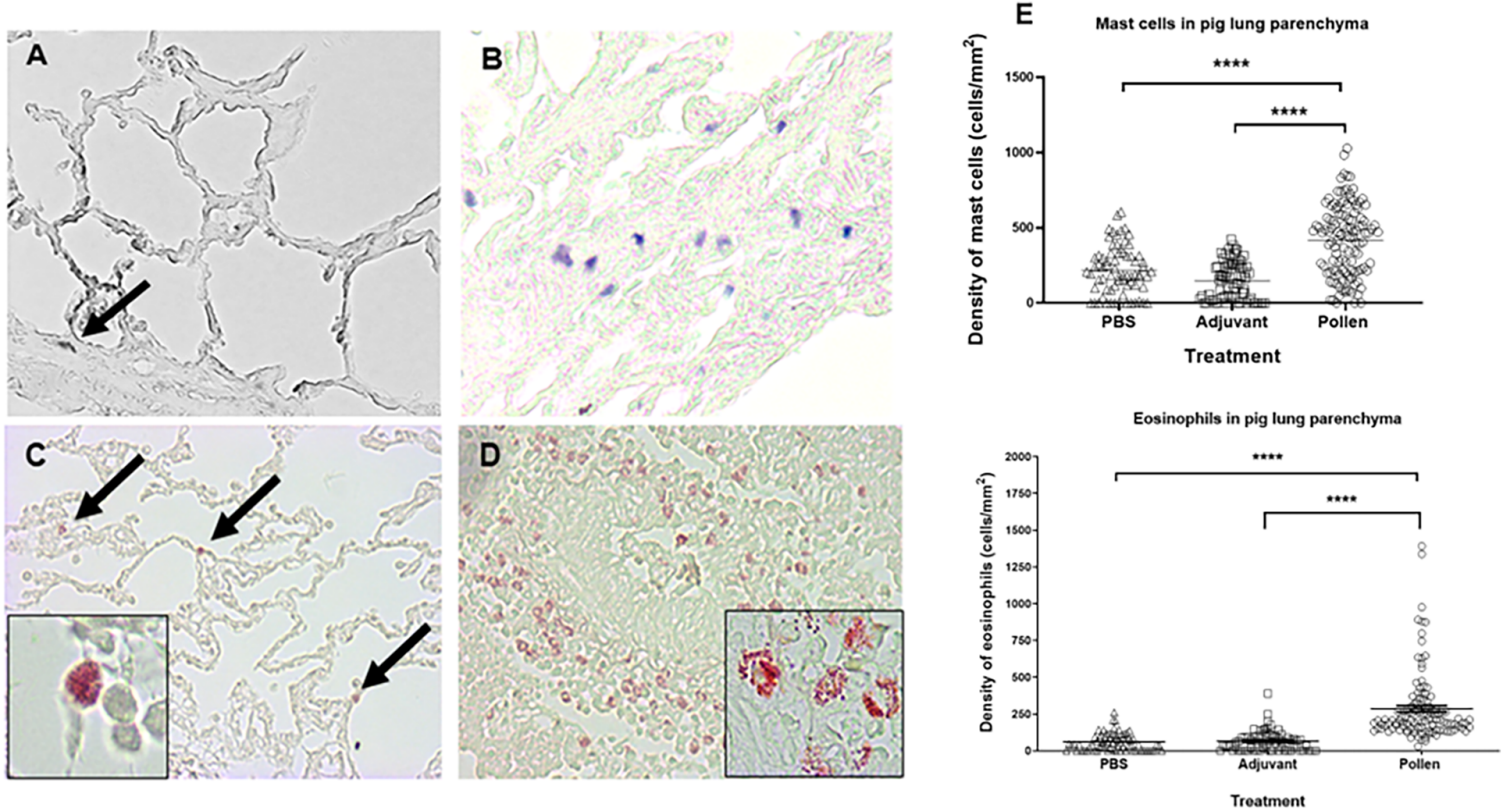
Histological images of lung parenchyma from control **(A,C)** and pollen sensitized **(B,D)** pigs (40X magnification). Toluidine blue mast cell staining **(A,B)** and chromotrope 2R neutrophil staining **(C,D)**. Insets (100x) show eosinophils in diverse stages of degranulation. **(E)** Density (cells/mm^2^) of mast cells and eosinophils in lung parenchyma. Each symbol represents the count of a microscopic field of at least 5 animals per group. Significance calculated by Unpaired t-test. ****p ≤ 0.0001.

Analysis of lung tissue from control pigs revealed a healthy structure of the bronchial parenchyma, well-defined alveolar spaces, and no damage (Figures 6A,C). Tissue from pigs treated with the pollen extract showed significant epithelial restructuring, bronchial congestion, increased mucus within the bronchi and decreased alveolar spaces (Figures 6B,D). In addition, degranulation of the infiltrate of both cell types was observed in the lung tissue of pollen-sensitized pigs (Figure 6D inset). The cell density was determined in each of the microscopic fields, calculating the total number of mast cells stained in blue-purple and eosinophils stained in red, per square millimeter of tissue. The area of bronchial epithelium and alveolar spaces were excluded to homogenize the count. Statistical analysis revealed a significantly increased infiltrate of these cells in the lung tissue of pollen-sensitized pigs compared to control pigs (Figure 6E).

### 3.6 Serum cytokine profile

**Figure 7:**
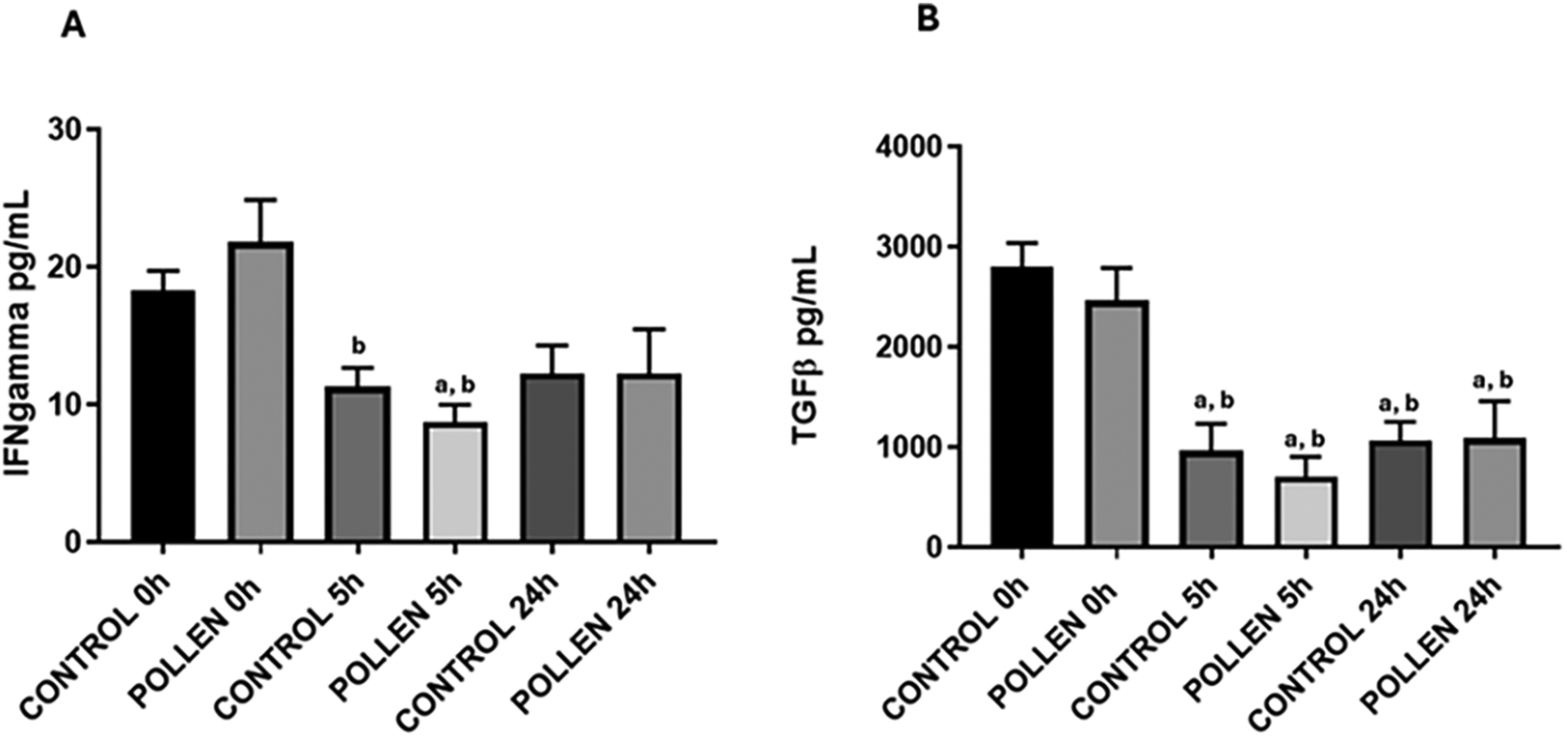
Serum IFNg **(A)** and TGFb **(B)** cytokine profile of control (*n* = 10) and sensitized (*n* = 11) pigs. Data expressed as mean ±SEM. Significance calculated by one-way ANOVA analysis, and Tukey's multiple analysis. ^a^*p* ≤ 0.05 vs. control 0 h, ^b^*p* ≤ 0.05 vs. pollen 0 h.

The cytokine response between pollen-sensitized and control pigs at 0, 5 and 24 h post-intratracheal challenge showed no significant difference in interleukins 4, 10, and 13 (data not shown) while for TGFβ and IFNγ, significant reductions were observed at various times (*p* ≤ 0.05) (Figure 7). In the case of IFN*γ*, the response decreased significantly at 5 h in both experimental groups concerning the time of 0 h (*p* ≤ 0.05) (Figure 7A), in TGFβ a significant decrease was found at 5 h and 24 h concerning the time of 0 h in both experimental groups (*p* ≤ 0.05) (Figure 7B).

### 3.7 Quantitative determination of allergen-specific IgA, IgG, and IgE

**Figure 8:**
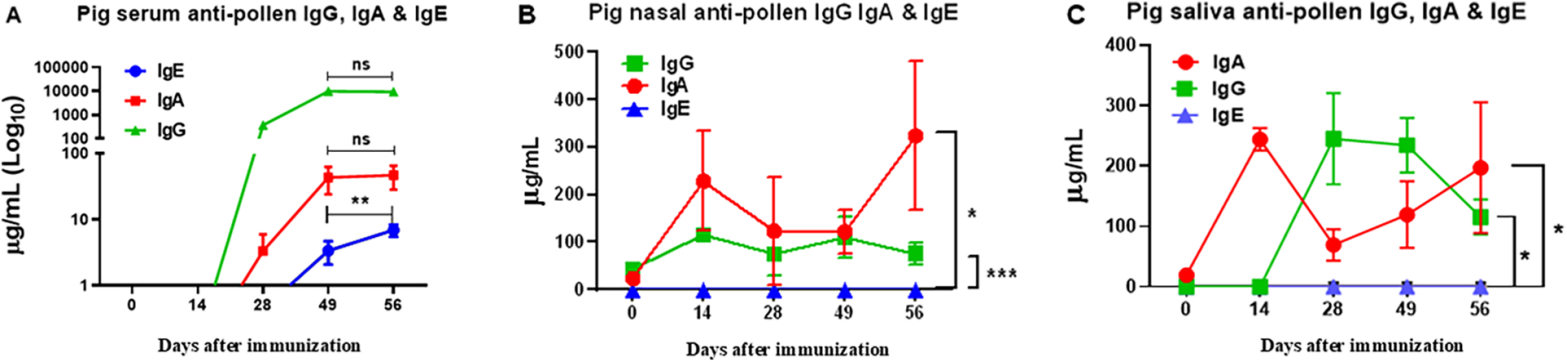
ELISA quantitative determination of allergen-specific IgA, IgG, and IgE in serum **(A)**, nasal **(B)**, and saliva **(C)** samples of pollen-sensitized pigs. Data expressed as mean ±SEM (*n* = 12). Significance calculated by one-way ANOVA analysis, and Tukey's multiple analysis. **p < 0.01 between dates in **(A)** *p ≤ 0.05 and ***p ≤ 0.001 vs. control groups in **(B,C)** Control groups were all negative (not shown). The graph scales are different.

Analysis of the production of different isotypes of specific antibodies against pollen proteins showed significant differences in serum, nasal mucus, and saliva between sensitized pigs and the control group. Only sensitized pigs showed anti-pollen IgG and IgA antibodies in serum reaching a maximum at day 49 post-immunization. Serum IgE was detected at low levels until day 49, but, in contrast to IgG and IgA, its concentration kept rising over time (Figure 8A). In nasal mucus and saliva samples, a significant difference was observed in the production of IgA and IgG antibodies in sensitized pigs compared to the control group. In contrast, IgE was not detected in these samples (Figures 8B,C).

## 4 Discussion

Asthma is a chronic disease that has a significant impact on the quality of life of the world population, particularly by its high incidence in children, adolescents, and older adults (41). The pig has great similarities with human anatomy and physiology, making it an experimental model that could provide information to complement the data obtained in more conventional models such as the mouse, rat, rabbit, and guinea pig (43), especially for pre-clinical studies.

The porcine respiratory tract has been accepted as a translational model for respiratory research (16–18), which offers the advantage of deepening studies in public health problems such as asthma and respiratory allergies. The bronchial structure and ventilatory patterns of pigs allow assessment of lung function with clinical techniques applicable to humans, such as spirometry and bronchoscopy. Furthermore, allergen-induced airway inflammation and remodeling in pigs more accurately reflect the human asthmatic phenotype compared to rodents (16, 19). Although their cost and handling are higher than murine models, this is compensated for the quantity and quality of the data obtained, as the porcine model provides more detailed information due to its higher biological complexity. It also allows for more advanced techniques and the collection of larger sample volumes, which improves the reproducibility and depth of the analyses.

Currently, this is the first study to establish a model of respiratory hypersensitivity in Vietnamese pigs and our findings provide an initial basis for future research on the applicability of this model in allergy studies. Therefore, in this study a novel immunization protocol was carried out in our experimental model (Vietnamese mini-pig), using *Phleum pratense* allergenic pollen extract, to induce a type-I-like respiratory hypersensitivity, to provide a model to future investigate the onset, development, diagnosis, treatment and prevention of such pathology. In our model, the intradermal reaction demonstrated an antigen-specific IgE-mediated response. This phenomenon is due to the degranulation of mast cells, which, during the process of immunization with *Phleum pratense* pollen, became sensitized with specific IgE against the pollen antigens, which bind to their high-affinity receptors on the mast cell membrane. Thus, when the test is performed and the antigen is administered, it interacts between two adjacent IgE, causing receptors cross-linking and, consequently, activation and degranulation of mast cells, releasing proinflammatory mediators that generate erythema and induration, as observed in our model. Moreover, these results are consistent with what has been reported in animal models with food allergy and intradermal tests used for allergy diagnosis in humans (44–47).

On the other hand, passive cutaneous anaphylaxis is a classic test used to confirm antigen-specific IgE-mediated responses. Heat inactivation allows the identification of the nature of the antibody involved. In our work, fresh sera from sensitized pigs gave a positive skin reaction, but a negative response was seen when these sera were heat-inactivated (Figure 3), confirming that an anti-*Phleum pratense* IgE mediated the response (47, 48). In the intratracheal challenge, clinical signs were highly variable in the porcine model as in an open human population. However, it was possible to discern between the effects caused by pollen instillation in control pigs (pulmonary edema only) and pollen-sensitized pigs, in which an exacerbated reaction was obtained upon challenge, with a series of clinical manifestations corresponding to anaphylactic shock (49, 50) (Table 1). The ability to observe and measure such clinical signs in pigs offers a significant advantage over rodent models, where these signs, particularly those indicative of severe allergic reactions, can be difficult to quantify or manifest less reliably (51, 52). For example, in rodents, anaphylactic reactions often require invasive procedures to accurately measure vital signs, which can be challenging or ethically controversial. In contrast, the porcine model allows non-invasive and more accurate real-time monitoring of clinical parameters such as heart rate, respiratory rate and blood pressure, which are critical for assessing the severity of anaphylactic reactions (53, 54). This model therefore not only provides a more robust platform for assessing allergic responses but also reflects the clinical complexity of anaphylaxis in humans. Hence, in some cases, drug administration was necessary to prevent fatal consequences, as in humans with respiratory anaphylactic shock.

Several studies have demonstrated changes in tissue architecture in lung biopsies from asthmatic individuals, like those observed in our experimental model. These structural changes are a consequence of the inflammatory process caused by pollen hypersensitivity, which has been reported as part of the pathophysiology of asthma, leading to tissue remodeling and an increase in inflammatory cells (mast cells, lymphocytes and eosinophils) (22, 55, 56). As observed in BAL cytology and lung histology, eosinophil infiltration indicated a quick response caused by specific pollen allergens. In addition, increased degranulated eosinophils were observed, characteristic of patients with allergic asthma and in established mouse models of asthma, where chronic bronchopulmonary inflammation with an abundant infiltrate of mast cells and eosinophils, localized even at the tissue epithelium is often found (57). This is closely associated with severe forms of asthma (55, 56, 58).

The study of inflammatory and anti-inflammatory cytokines is of great relevance for understanding immune responses. In the case of IL-4, IL-6, IL-10, and IL-13 levels in serum samples, no significant differences were observed between the control and treated groups. This finding is consistent with previous studies that evaluated the prevalence of the Th1 and Th2 cytokine profiles in serum samples from individuals with stable asthma (59–61). Similarly, other authors have reported that elevated cytokine levels only occur under specific conditions, such as during chronic phases of asthma accompanied by bronchial hyperresponsiveness, prior to receiving pharmacological treatment (46, 55, 59). On the other hand, significant changes in TGF-β and IFN-γ levels were observed with respect to the time of immunization, but not between the treated and control groups. These findings are consistent with previous studies, for example, in childhood populations with asthma and risk factors such as obesity, where it has been reported that modulators of the immune response, such as serum IFN-γ, do not show significant differences compared to non-overweight individuals (62). A similar phenomenon has been described in pregnant women with and without asthma (63).

Metabolomic studies have also evaluated serum TGF-β and IFN-γ levels in patients with chronic obstructive respiratory disease (COPD) and in those with both COPD and asthma, compared to smokers. These cytokines were found to be significantly altered in patients with COPD and asthma, but not in those with asthma or in smokers (64). TGF-β is closely related to structural changes in the respiratory epithelium and fibrotic processes, so its increase depends on disease severity and lung function impairment (65–67). Finally, an increase in serum TGF-β levels has been associated with irreversible processes of pulmonary fibrosis in asthmatics, and IFN-γ has been described to have an immunomodulatory effect. A decrease in this cytokine during acute episodes has been reported in asthmatics. It is likely that IFN-γ binds to cellular receptors during inflammatory processes to exert its anti-inflammatory function (68, 69).

In asthma, the kinetics of the appearance of immunoglobulins IgA, IgG, and IgE play a fundamental role in modulating the immune response. The IgE, central to the allergic response, is rapidly activated upon allergen exposure, with levels peaking after approximately 14 days (70). This elevation triggers mast cell and basophil degranulation, promoting airway inflammation (50). We measured the antigen-specific IgE, IgA, and IgG, providing insights into the sequential immune dynamics. The initial IgG response may serve to moderate inflammation, functioning as a regulatory mechanism to limit antigen entrance and excessive hypersensitivity (71). Moreover, IgA primarily operates at the mucosal level, neutralizing inhaled allergens and pathogens, thus providing a protective barrier, as observed during pollen instillations in the porcine tonsil (72). Our results indicate that, although serum IgG and IgA responses increased initially to prevent sensitization potentially, these responses plateau after approximately five to seven weeks, coinciding with the late appearance of allergen-specific IgE, albeit at low serum concentrations (Figure 8A). Given IgE's high cytotropic nature, it may bind predominantly to mast cell membranes, with concentrations rising over time increasing the chances of a hypersensitivity reaction. Further long-term studies are needed to understand these immunoglobulin kinetics and their relationship to cytokine dynamics, particularly in chronic models of respiratory hypersensitivity. Therefore, our model may offer valuable insights into the onset, progression, and regulation of the humoral immune response in respiratory hypersensitivity mediated by IgE, giving clues for future treatments.

The porcine model has multiple advantages over other animal models, among them: its high genome and protein sequence homologies with humans, its close resemblance of human immune parameters (>80% vs. <10% for mice), many tools are available (cell lines, antibodies, ELISA and microarrays), cloning and transgenic technology advances, its docility, relatively short gestational and developmental periods, numerous offspring, monogastric and omnivorous nature, size and weight close to the human allowing translational results for treatments, and cheaper and ethically more acceptable use than primates (15–17). These advantages might outweigh the cost of animals, feed and housing in pre-clinical studies for pathologies as important as asthma. In our hands, we could recognize clinical asthma signs like those in humans, and the sample size was large enough for more comprehensive studies (BAL, histology, mucosal fluids).

## 5 Conclusions

Our immunization protocol in the swine model induced a respiratory hypersensitivity type I-like reaction against *Phleum pratense* antigens mediated by antigen-specific IgE. The model also showed clinical and histopathological similarities in skin and lungs, and the production of antibodies, like human physiopathology in the face of the allergenic challenge with a complex allergen such as *Phleum pratense* pollen. It would be necessary to establish a chronic state of the disease using a longer protocol in the swine model, to study the molecular mechanisms involved in the beginning, development, and establishment of the condition resembling human asthma. This model will help in pre-clinical studies to develop early diagnostic tools and therapeutic and prophylactic measures to treat this pathology.

## Data Availability

The raw data supporting the conclusions of this article will be made available by the authors, without undue reservation.
